# Effect of coffee intake on hip fracture: a meta-analysis of prospective cohort studies

**DOI:** 10.1186/s12937-015-0025-0

**Published:** 2015-04-18

**Authors:** Shuai Li, Zhipeng Dai, Qiang Wu

**Affiliations:** Department of Orthopedics, Union Hospital, Tongji Medical College, Huazhong University of Science and Technology, 1277 Jiefang Avenue, Wuhan, 430022 P. R. China

**Keywords:** Coffee consumption, Hip fracture, Meta-analysis, Prospective cohort studies

## Abstract

Several observational studies suggest an association between coffee intake and hip fracture risk. However, the results among them are inconsistent. We aimed to evaluate the association between coffee consumption and risk of hip fracture by performing a meta-analysis of prospective cohort studies. PubMed, Embase, and Web of Science were searched through July 2014 to identify studies that met pre-stated inclusion criterion and reference lists of retrieved articles were reviewed. Information on the characteristics of the included study, risk estimates, and control for possible confounding factors were extracted independently by two authors. A random effects model of meta-analysis was used to calculate the pooled risk estimate. Ten prospective cohort studies involving 5408 patients with hip fracture and 205,930 participants were included in this systematic review. Compared with individuals who did not or seldom drink coffee, the pooled relative risks of hip fracture was 1.13 (95% confidence interval: 0.86 to 1.48) for individuals with the highest coffee consumption. Exception of any single study did not materially alter the combined risk estimate. Visual inspection of funnel plot and Begg’s and Egger’s tests did not indicate evidence of publication bias. In summary, integrated evidence from prospective cohort studies does not suggest a statistically significant association between coffee consumption and risk of hip fracture in developed countries.

## Introduction

Hip fracture, one of the most serious complications of osteoporosis, has become a major health problem in many countries in recent decades because of the rapid increase in incidence. It is estimated that there could be 4.5 million hip fractures by 2050 [1]. Identifying and confirming the modifiable risk factors and protective factors of hip fracture incidence is of significant importance for developing preventive strategies. The potential influence factors for hip fractures include daily calcium intake [2], physical activity level [3,4], body mass index (BMI) [5,6], smoking [7], and alcohol consumption [8].

Coffee, the main source of dietary caffeine intake [9,10], is one of the most widely consumed beverages in the world. Epidemiological studies have, however, found that high dietary caffeine intake is associated with reduced bone mineral density and increased body calcium loss [11,12]. An earlier meta-analysis by Liu et al. [13] suggested that coffee consumption has an overall harmful effect of increasing the risk of fractures. However, they did not provide the results of the subtypes of fractures, which may have reduced the strength of their conclusions. Obviously, it is very possible that coffee has different effect on different subtypes of fractures, but that meta-analysis did not detail it. Identifying the association of coffee with specific fracture including hip fracture may have more significant value to clinician and the general public. Although there have been some population-based observational epidemiological studies [8,14-21] on the relationship between coffee intake and hip fracture since the 1990s, the finding have been inconsistent. Recently a meta-analysis of observational studies [22] was conducted to evaluate the association between coffee consumption and hip fracture and concluded that there was no significant association between coffee consumption and the risk of hip fracture. However, the literature searching of that meta-analysis was not comprehensive. It only included six cohort studies, but three other eligible prospective cohort studies [14,15,21], which suggested inconsistent relationship between coffee intake and hip fracture risk, were missing. In addition, although heterogeneity among cohort studies was substantial, the source was not identified adequately. Given that coffee is consumed very commonly all over the world, an improved understanding of this issue should have important public health and clinical implications.

Taking into consideration the inconsistent conclusions of existing epidemiological studies and the higher level of evidence from prospective cohort studies, along with three missing studies in the previous meta-analysis, we performed an updated meta-analysis of nine prospective cohort studies to evaluate the association between coffee consumption and the risk of hip fracture.

## Methods

### Literature search

We conducted this systematic review in accordance with the meta-analysis of observational studies in epidemiology guidelines [23]. A systematic literature search of PubMed, Embase, and Web of Science was conducted through July 2014 by using the following search terms with no restrictions: “coffee” in combination with “fracture”. Reference lists of the retrieved articles were also reviewed. We did not contact authors of the primary studies for additional information.

### Study selection

We included studies which met the following criteria: (1) it had a prospective cohort study design; (2) the exposure of interest was consumption of coffee; (3) the endpoint of interest was incidence of hip fracture; (4) the relative risk (RR) and the corresponding 95% confidence interval (CI) of hip fracture relating to the total or to all categories of coffee intake were reported or could be calculated from the data provided; and (5) the frequency and dose of coffee consumption were provided. Studies were excluded if mixed beverages were reported in which the effect of coffee could not be desolated. If multiple published reports were from the same study cohort, we included only the one with the most detailed information for both coffee consumption and outcome.

### Data extraction

The following information was extracted from the studies included in the final analysis by two investigators: first author, publication year, country, study period, number of cases, size of cohort, adjusted RR with 95% CI, and adjusted factors. Discrepancies were resolved by discussion with a third investigator.

### Statistical analyses

We used RR to measure the association of interest. In any included study, the lowest level of coffee consumption was defined as “never or seldom drink coffee” and was treated as reference. The exposure group was the individuals with the highest level of coffee intake. We calculated an overall pooled RR using a random effects model for the main analysis [24]. Heterogeneity was tested by *Q* statistic with a significance level at *P* < 0.10 and *I*^2^ statistic [25]. The *I*^2^ statistic measures the percentage of total variation across studies due to heterogeneity rather than chance. It is calculated according to the following formula:

$$ {\mathrm{I}}^2=100\%\times \frac{\mathrm{Q}-\mathrm{d}.\mathrm{f}.}{\mathrm{Q}}, $$

where Q is the heterogeneity statistic, and d.f. is the degree of freedom. The negative value of *I*^2^ is set at zero, and *I*^2^ varies from 0% (no observed heterogeneity) to 100% (maximal heterogeneity). An *I*^2^ value of ≥50% is considered to represent substantial heterogeneity.

Subgroup analyses were conducted to determine the possible influence of some factors such as publication years, countries, length of follow-up, and calcium intake. A sensitivity analysis was conducted to explore potential sources of heterogeneity and to investigate the influence of various exclusion criteria on the pooled risk estimate. Potential publication bias was assessed by both the Begg rank correlation and the Egger linear regression tests [26,27]. All analyses were conducted using STATA statistical software (version 12.0; College Station, TX, USA). P < 0.05 was considered statistically significant, except where otherwise specified. All statistical tests were two-sided.

### Ethical approval

Ethical approval is not required for this review.

## Results

### Literature search

**Figure 1:**
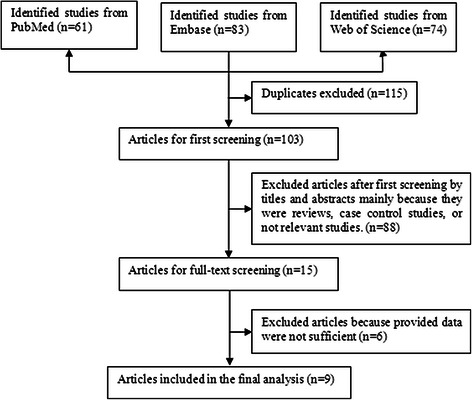
Flow diagram of literature search and study selection.

The process of study identification is shown in Figure 1. Initially we retrieved 61 citations from the PubMed database, 83 citations from Embase, and 74 citations from Web of science. After 115 duplicates were excluded, 103 citations were screened through titles and abstracts, 88 of them were excluded mainly because they were case–control studies, cross-sectional studies, reviews, or irrelevant studies. After full-text review of the remaining 15 articles, six articles were excluded because they did not report RRs and the corresponding 95% CI of interest or provide sufficient data to calculate them. Of the remaining articles, one article [20] reported its results separately by sex, and was regarded as two separate studies. Finally, nine articles involving ten studies were included.

### Study characteristics

The main characteristics of the ten prospective cohort studies are presented in Table 1. These studies were published between 1991 and 2013. Of these studies, five were conducted in the United States, while the remaining five studies were conducted in four European countries (one in Finland, Netherland, Sweden, and two in Norway). Sample sizes ranged from 1,222 to 84, 484 (total 205, 930). Measurement of coffee consumption was obtained by questionnaires in all the included studies, and the ascertainment of hip fracture was based on medical record, self-report, or radiological confirmation. Estimation of RRs was adjusted in all studies, but the number of adjusted variables varied across studies.

**Table 1:** **Characteristics of included studies of coffee in relation to risk of hip fracture**

Author	Year	Country	Period	Duration (years)	Cases	Participants	Mean age or age range (years)	Assessment of coffee consumption	Hip fracture ascertainment	Highest vs. lowest category of coffee consumption	RR (95% CI)	Adjustments
Cummings S R	1995	America	1986-1990	4	192	9516	≥65	Frequency questionaire	Radographs	≥1 vs. never	1.20 (1.00 -1.50)	Fractures and calcaneal bone density
Hallstrom H	2013	America	1987-2008	22	3871	14738	≥50	Frequency questionaire	Hospital registers	≥4 vs. < 1	0.88 (0.78 -1.00)	Age, body mass index, height, total energy intake, and dietary intakes of calcium, vitamin D, retinol, protein, phosphorous, potassium, and alcohol, vitamin D supplementation, tea consumption, educational level, physical activity level, smoking status, previous fracture, Charlson comorbidity index, living condition (living alone or not), nulliparity, cortisone use, and hormone replacement therapy.
Hansen S A	2000	America	1986-1992	6.5	275	34703	55-69	Frequency questionaire	Self-reportd	≥4 vs. ≤ 0.5	0.92 (0.62 -1.36)	Age, alcohol intake, calcium intake, estrogen-replacement therapy, smoking, physical activity, body mass index, caloric intake, and waist hip rate
Hernandez-Avila M	1991	America	1980-1986	6	59	84484	34-59	Frequency questionaire	Self-reported	≥4 vs.never	3.35 (1.32 -8.49)	Age, Quetelet index, menopause status, oestrogen-replacement therapy, calcium intake, alcohol intake
Jokinen H	2010	Finland	1997-2007	10	21	1222	72	Frequency questionaire	Medical records	>5 vs. <5	2.58 (1.01 -6.56)	Body mass index, physical activity, calcium intake, smoking, alcohol use, and cardiovascular disease.
Kiel D	1990	America	1971-1982	12	135	17,256	76.5	Frequency questionaire	Medical records and self-reported	>2 vs. ≤ 2	1.82 (1.09 -3.05)	Sex, age, Framingham examination number, metropolitan relative weight, postmenopausal oestrogen use, smoking, alcohol consumption
Meyer H E	1997	Norway	1977-1991	11.4	212	19938	56.5	Frequency questionaire	Medical records or discharge letters	≥9 vs. <2	1.94(0.96 -3.91)	Age, body height, body mass index, self-reported physical activity at work and during leisure time, diabetes mellitus, disability pension, marital status, and smoking
											for women	
											1.04 ( 0.37 -2.94)	
											for men	
Trimpou P	2010	Sweden	1974-2003	30	451	7495	46-56	Frequency questionaire	Hospital discharge register	>5 vs.never	0.55 (0.42 -0.73)	Age, physical activity, smoking, stature, occupation, alcohol abuse, intercurrent stroke, dementia, serum cholesterol concentrations, body mass index, diabetes mellitus, psychological stress, systolic blood pressure
van Lenthe F J	2011	Netherlands	1991-2003	13	192	16578	>55	Frequency questionaire	Hospital admission database	≥3 vs. never	0.75 (0.42 -1.36)	Father’s occupation, adult occupation, education, income proxy

### Main results of meta-analysis

**Figure 2:**
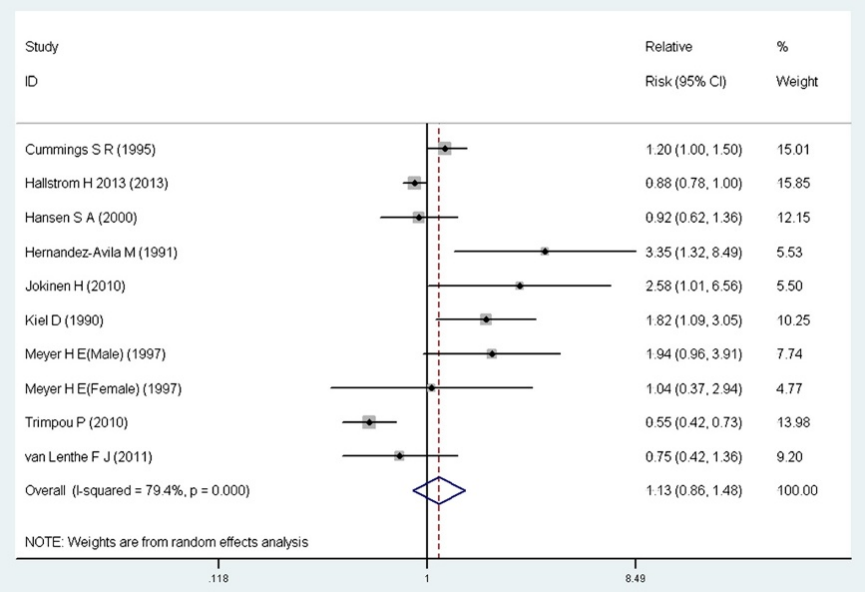
Association between coffee consumption and the risk of hip fracture in a meta-analysis of prospective cohort studies.

Figure 2 showed the results from the random-effects model combining the risk estimates. Of the ten studies, three showed a significantly positive relationship between coffee consumption and the risk of hip fracture, and six suggested no statistically significant association of interest. But the remaining one gave a reverse conclusion of the relationship. Overall, compared with individuals who seldom or never drink coffee, the pooled RR of hip fracture was 1.13 (95% CI: 0.86 to 1.48) for individuals with the highest level of coffee consumption. Statistically significant heterogeneity was observed across the included studies (*p* = 0.000, *I*^2^ = 79.4%).

### Subgroup analysis and sensitivity analysis

In order to identify the possible source of heterogeneity and the potential difference in results among various subgroups, we performed subgroup analysis. Subgroup analysis by region (European countries vs. USA) showed no statistically significant difference in results (Table 2). We performed subgroup analysis by publication years (before 2000 vs. after 2000) and found that, although no significant association of interest was identified based on studies after 2000, the studies published before 2000 showed a positive relationship between coffee intake and hip fracture risk (RR: 1.58; 95% CI: 1.11 to 2.24). Given the possible influence of calcium intake on the association between coffee and hip fracture risk, we also conducted subgroup analyses by whether it was controlled. In the results, whether calcium intake was adjusted or not, the association did not change. Subgroup analysis by sex and length of follow-up both suggested no significant difference in results. We excluded any single study in turn and pooled the results of the remaining included studies to evaluate the stability of the results. The overall combined RR did not materially change, with a range from 1.05 (95% CI: 0.81 to 1.36) to 1.24 (95% CI: 0.96 to 1.61).

**Table 2:** **Subgroup analysis of relative risk of coffee intake for hip fracture**

	Number of studies	Relative risk	95% confidence intervals	I-square	P for heterogeneity
**Countries**					
European countries	5	1.08	0.59 to 2.00	78.9%	0.001
USA	5	1.20	0.89 to 1.62	79.0%	0.001
**Publication years**					
Before 2000	5	1.58	1.11 to 2.24	46.8%	0.111
After 2000	5	0.84	0.62 to 1.14	74.0%	0.004
**Sex**					
Male	2	0.53	0.38 to 1.00	26.2%	0.244
Female	6	1.27	0.94 to 1.72	76.4%	0.001
Both	2	1.19	0.50 to 2.82	79.8%	0.026
**Adjusted for calcium intake**					
Yes	4	1.31	0.81 to 2.11	76.1%	0.006
No	6	1.07	0.69 to 1.66	83.7%	0.000
**Length of follow-up (years)**					
<10	3	1.28	0.82 to 1.98	68.7%	0.041
10-20	5	0.71	0.45 to 1.12	89.2%	0.002
>20	2	1.46	0.92 to 2.29	50.6%	0.088

### Publication bias

Visual inspection of a funnel plot failed to identify substantial asymmetry. The Begg rank correlation test and Egger linear regression test also indicated no evidence of publication bias among the studies (Begg’s test Z = 0.89, *p* = 0.371; Egger’s test t = 1.47, *p* = 0.181).

## Discussion

Coffee is a widely consumed beverage around the world and previous studies and meta-analysis suggested that coffee consumption may affect the etiology of various diseases along multiple pathways. Roasted coffee is a complex mixture of more than one thousand chemicals, in which many constituents could potentially alter disease hazard through several biological mechanisms. Recently, in viro studies have suggested that caffeine deleteriously affects osteoblasts directly or indirectly. By increasing urinary calcium excretion and decreasing intestinal absorption efficiency of calcium, caffeine might contribute to bone loss. The association between coffee consumption and the risk of hip fracture has been of increasing interest to the general public recently. Though the potential mechanisms that how coffee impacts hip fracture risk have not been studied thoroughly, some epidemiological studies investigated the association between them. Our meta-analysis of ten prospectively cohort studies involving 205, 930 participants showed no significant association between coffee consumption and the risk of hip fracture.

A cohort study gives stronger evidence than a case–control study, and a retrospective cohort design may suffer more confounding factors and biases than a prospective one. This updated meta-analysis focusing on prospective cohort studies provided more robust evidence on the association between coffee intake and risk of hip fracture, and further confirmed the results of the previous meta-analysis of observational studies.

Taking into consideration the potential influence of countries in which the studies were conducted, sex, lengths of follow-up, publication years, and calcium intake, on the combined results, we performed corresponding subgroup analysis. Most subgroups did not showed different results except for publication years. The pooled results of five studies published before 2000 showed a positive relationship between coffee intake and hip fracture risk (RR: 1.58; 95% CI: 1.11 to 2.24). It seems details reported in the included studies did not give us an opportunity to explain this. Therefore, the specific reason of it was uncertain.

Of the included ten prospective cohort studies, Only one study adjusted vitamin D and vitamin D supplementation when the effect of coffee intake on hip fracture was analyzed quantificationally [15], and in that study the estimate of association between coffee intake and hip fracture was of borderline significance (OR 0.88; 95% CI 0.78 -1.00). Since most of included studies did not examined the potential influence of vitamin D on the association between coffee intake and hip fracture, we thought whether there was correlation between vitamin D levels to hip fracture and coffee consumption was inconclusive. To investigate this issue, further studies should be conducted. Since there is a relationship between caffeine levels and osteoblast activity, some researchers might be wondering if there was an effect of coffee intake on hip fracture in post-menopausal women. Although most of the included studies of our meta-analysis failed to report whether they involved post-menopausal women, Mean ages or age ranges of participants (see Table 1) in the original studies indicated that post-menopausal women were involved. All included studies involved middle-aged and elderly people and participants in one of these studies were all post-menopausal women [16]. Therefore, our conclusion should be reliable not only in general people but also in post-menopausal women.

There are several strengths in our study. Firstly, all the included studies were prospective cohort studies which give stronger evidence than case–control studies and retrospective cohort studies. Secondly, large sample sizes (5408 patients with hip fracture and 205,930 participants) and consistent results from sensitivity analysis indicated that our findings were reliable and robust. In addition, publication bias was unlikely to account for our findings, as identified by visual inspection of a funnel plot, the Begg rank correlation test, and the Egger linear regression test.

Some limitations in the present meta-analysis should be of concern. Firstly, adjusted confounders varied among the included studies. Some possibly important residual confounders such as calcium intake, physical activity level, and smoking were not adjusted in some studies. Secondly, due to the limited information provided by the included studies, a dose–response analysis was not performed to provide further evidence. Thirdly, different data collection instruments for coffee, such as diet habit questionnaire, dietary recall history, and food frequency questionnaire, were used in different studies. The veracity of these devices was different, and lack of unity in the type of them might contribute to heterogeneity among studies. There were also no standardized assessments or measurements for the amounts of coffee consumption. In the included studies, coffee consumption was mostly assessed by the number of cups per day or per week. However, there were differences in coffee bean roasting and brewing methods, and coffee cup size among the included studies. Additionally, different ascertainment methods such as medical record, self-report, and radiological confirmation might also contribute to heterogeneity. Finally, all included studies were conducted in developed countries and the applicability of our result in developing studies is pendent. Recent years, there are increasing coffee drinkers in developing countries, but relevant studies are relatively few.

In summary, our meta-analysis of prospective cohort studies with the most up-to-date evidence does not suggest a statistically significant association between coffee consumption and risk of hip fracture. However due to insufficient data from developing countries this conclusion is only applicable for developed countries.
